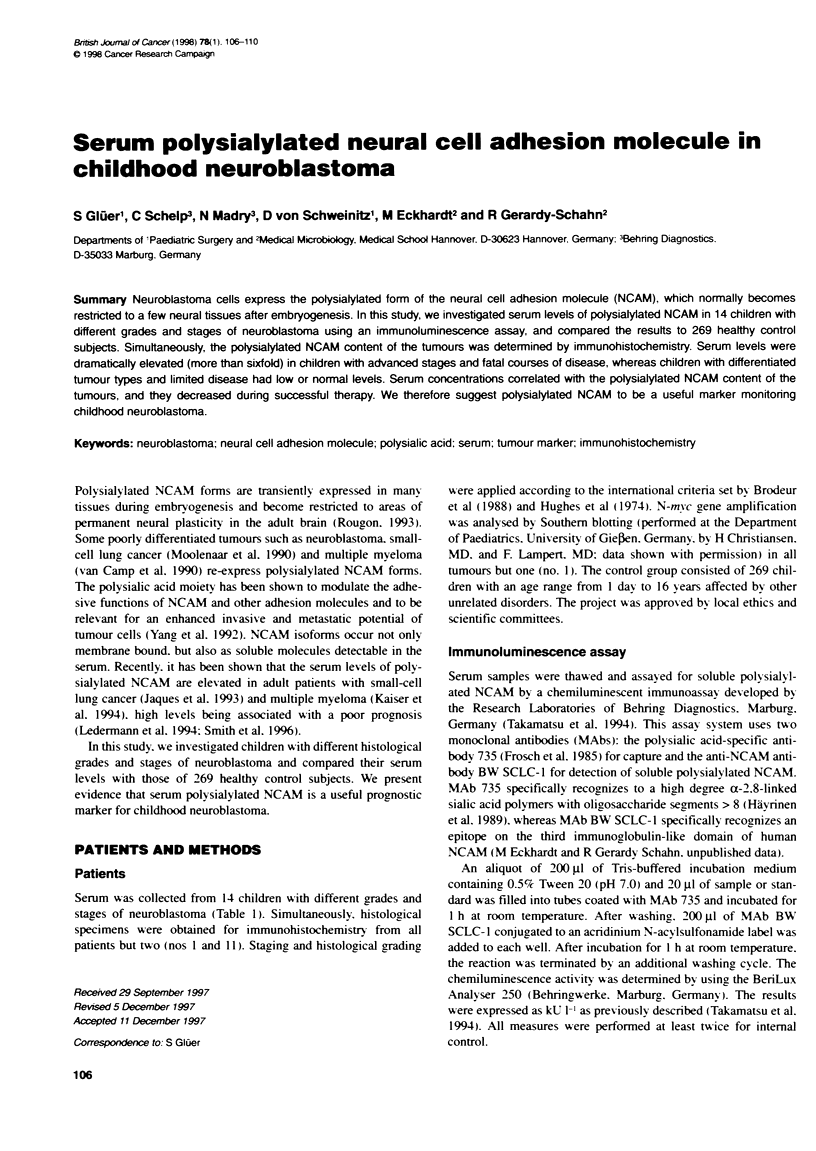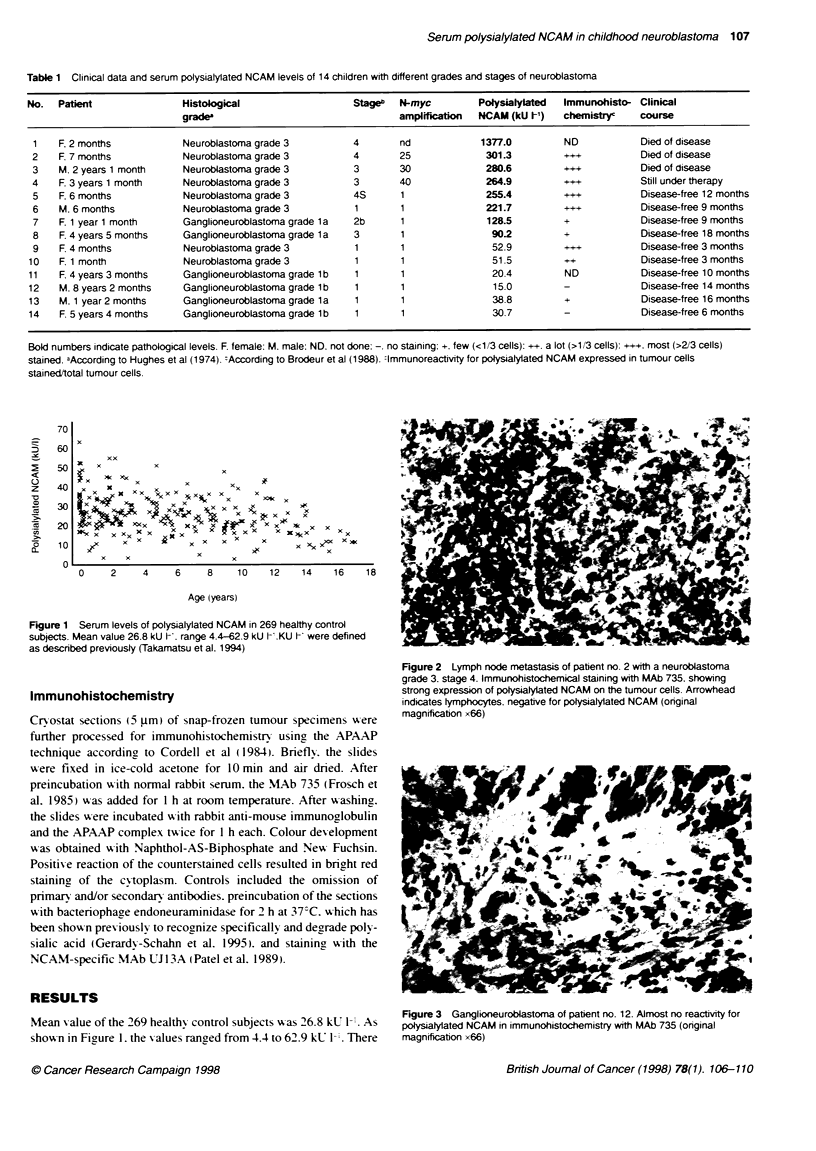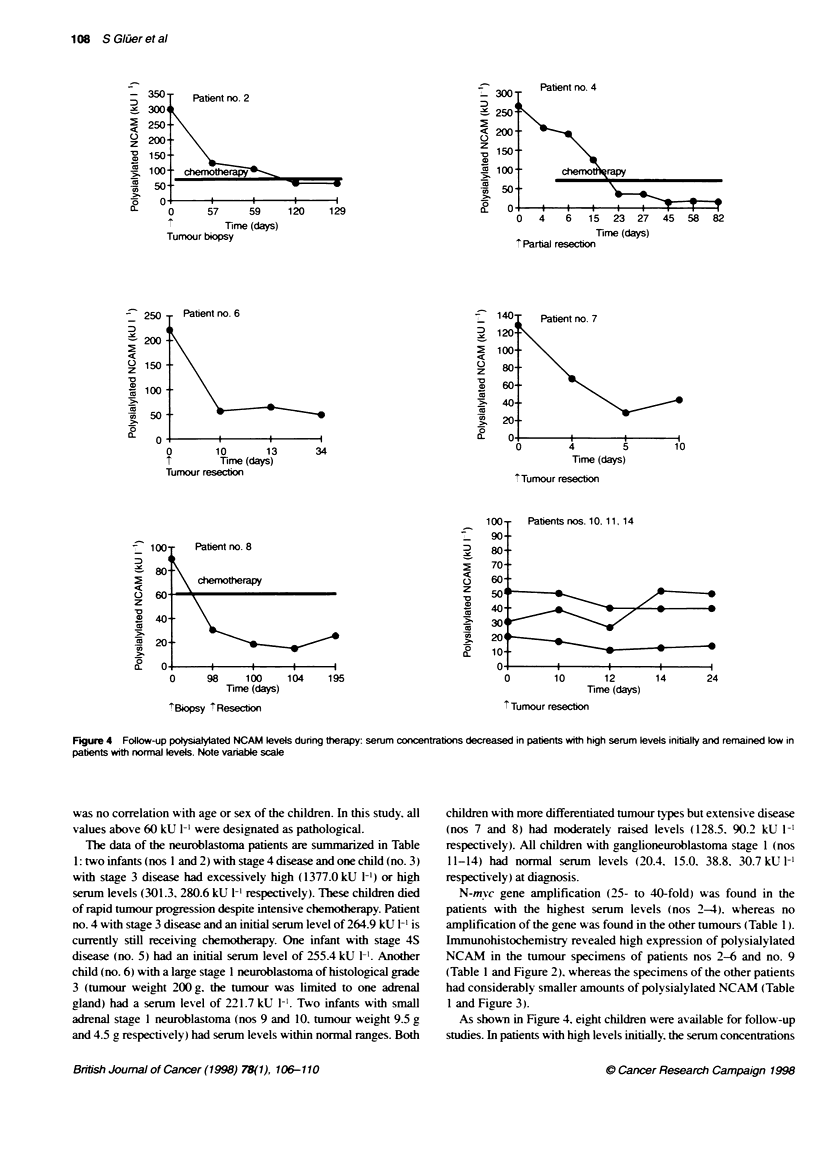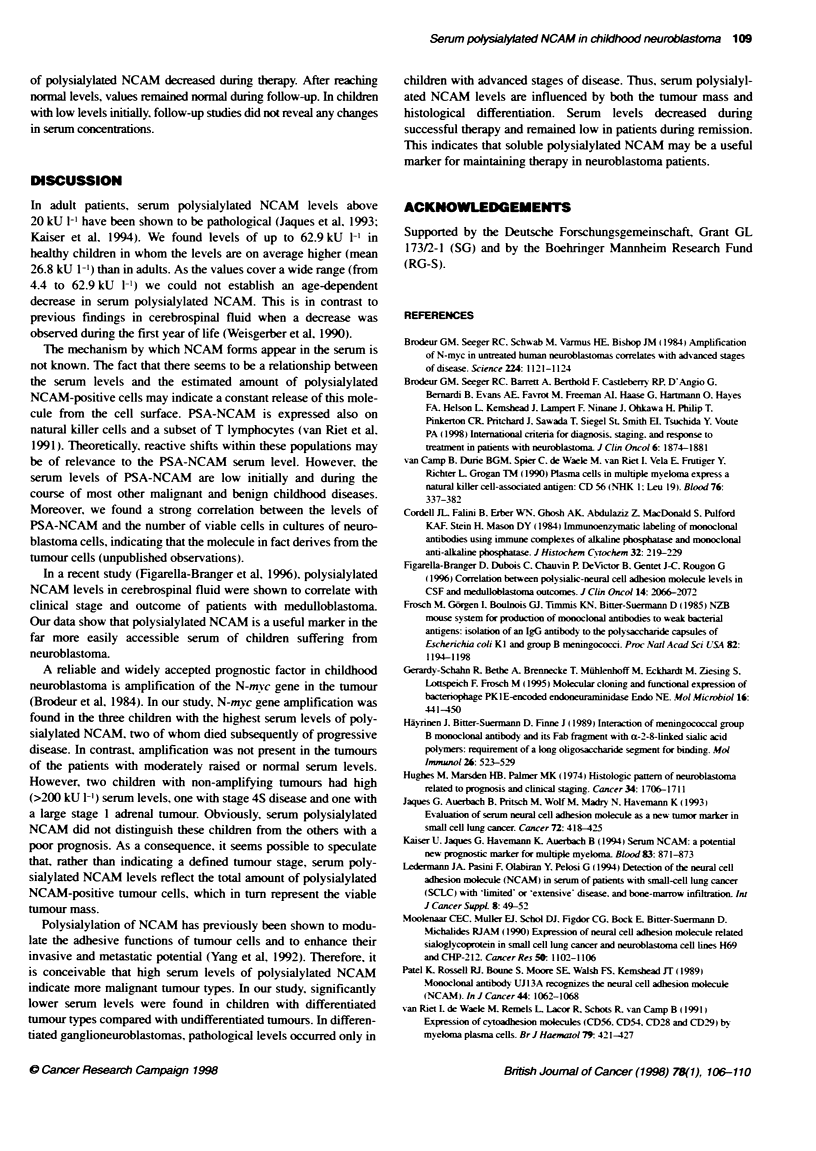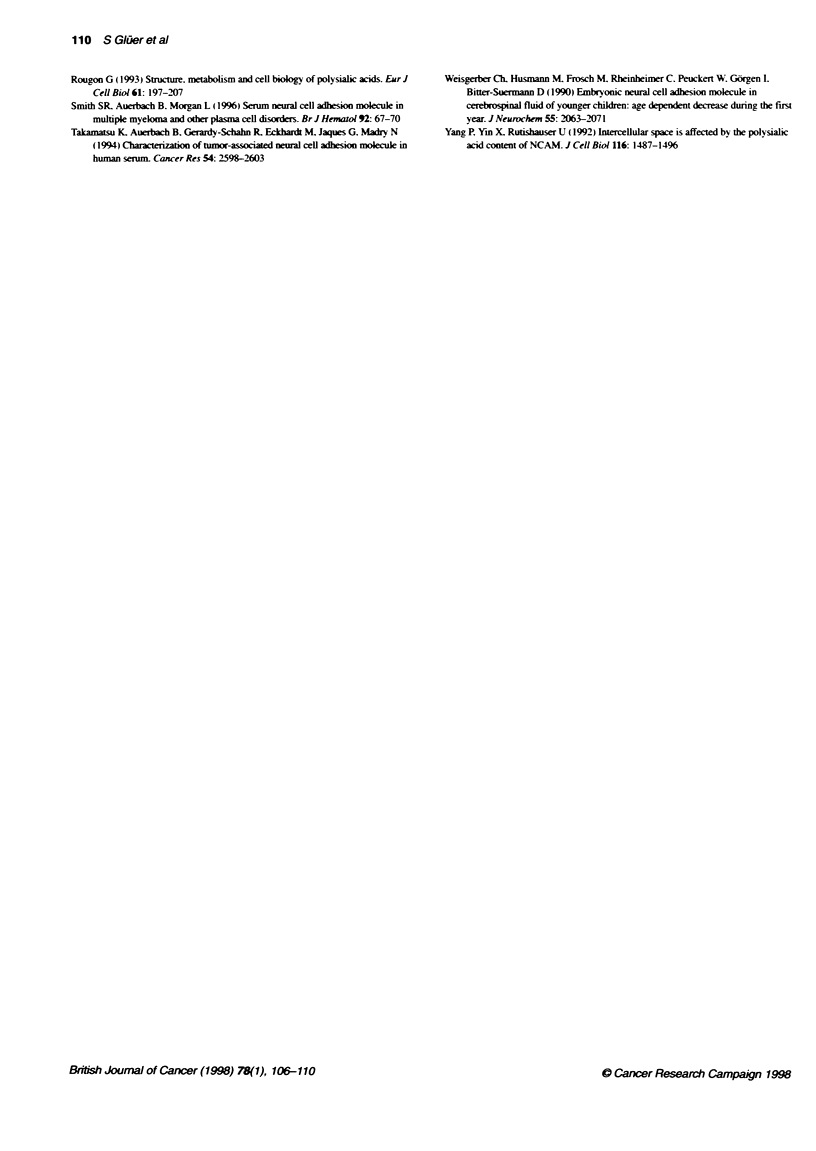# Serum polysialylated neural cell adhesion molecule in childhood neuroblastoma.

**DOI:** 10.1038/bjc.1998.450

**Published:** 1998-07

**Authors:** S. Glüer, C. Schelp, N. Madry, D. von Schweinitz, M. Eckhardt, R. Gerardy-Schahn

**Affiliations:** Department of Paediatric Surgery, Medical School Hannover, Germany.

## Abstract

**Images:**


British Journal of Cancer (1998) 78(1), 106–110
© 1998 Cancer Research Campaign

### Serum polysialylated neural cell adhesion molecule in childhood neuroblastoma

**S Glüer[1], C Schelp[3], N Madry[3], D von Schweinitz[1], M Eckhardt[2] and R Gerardy-Schahn[2]**

Departments of [1]Paediatric Surgery and [2]Medical Microbiology, Medical School Hannover, D-30623 Hannover, Germany; [3]Behring Diagnostics, D-35033 Marburg, Germany

**Summary** Neuroblastoma cells express the polysialylated form of the neural cell adhesion molecule (NCAM), which normally becomes restricted to a few neural tissues after embryogenesis. In this study, we investigated serum levels of polysialylated NCAM in 14 children with different grades and stages of neuroblastoma using an immunoluminescence assay, and compared the results to 269 healthy control subjects. Simultaneously, the polysialylated NCAM content of the tumours was determined by immunohistochemistry. Serum levels were dramatically elevated (more than sixfold) in children with advanced stages and fatal courses of disease, whereas children with differentiated tumour types and limited disease had low or normal levels. Serum concentrations correlated with the polysialylated NCAM content of the tumours, and they decreased during successful therapy. We therefore suggest polysialylated NCAM to be a useful marker monitoring childhood neuroblastoma.

**Keywords:** neuroblastoma; neural cell adhesion molecule; polysialic acid; serum; tumour marker; immunohistochemistry

Polysialylated NCAM forms are transiently expressed in many tissues during embryogenesis and become restricted to areas of permanent neural plasticity in the adult brain (Rougon, 1993). Some poorly differentiated tumours such as neuroblastoma, small-cell lung cancer (Moolenaar et al, 1990) and multiple myeloma (van Camp et al, 1990) re-express polysialylated NCAM forms. The polysialic acid moiety has been shown to modulate the adhesive functions of NCAM and other adhesion molecules and to be relevant for an enhanced invasive and metastatic potential of tumour cells (Yang et al, 1992). NCAM isoforms occur not only membrane bound, but also as soluble molecules detectable in the serum. Recently, it has been shown that the serum levels of polysialylated NCAM are elevated in adult patients with small-cell lung cancer (Jaques et al, 1993) and multiple myeloma (Kaiser et al, 1994), high levels being associated with a poor prognosis (Ledermann et al, 1994; Smith et al, 1996).

In this study, we investigated children with different histological grades and stages of neuroblastoma and compared their serum levels with those of 269 healthy control subjects. We present evidence that serum polysialylated NCAM is a useful prognostic marker for childhood neuroblastoma.

### PATIENTS AND METHODS

### Patients

Serum was collected from 14 children with different grades and stages of neuroblastoma (Table 1). Simultaneously, histological specimens were obtained for immunohistochemistry from all patients but two (nos 1 and 11). Staging and histological grading were applied according to the international criteria set by Brodeur et al (1988) and Hughes et al (1974). N-*myc* gene amplification was analysed by Southern blotting (performed at the Department of Paediatrics, University of Gießen, Germany, by H Christiansen, MD, and F. Lampert, MD; data shown with permission) in all tumours but one (no. 1). The control group consisted of 269 children with an age range from 1 day to 16 years affected by other unrelated disorders. The project was approved by local ethics and scientific committees.

### Immunoluminescence assay

Serum samples were thawed and assayed for soluble polysialylated NCAM by a chemiluminescent immunoassay developed by the Research Laboratories of Behring Diagnostics, Marburg, Germany (Takamatsu et al, 1994). This assay system uses two monoclonal antibodies (MAbs): the polysialic acid-specific antibody 735 (Frosch et al, 1985) for capture and the anti-NCAM antibody BW SCLC-1 for detection of soluble polysialylated NCAM. MAb 735 specifically recognizes to a high degree α-2,8-linked sialic acid polymers with oligosaccharide segments > 8 (Häyrinen et al, 1989), whereas MAb BW SCLC-1 specifically recognizes an epitope on the third immunoglobulin-like domain of human NCAM (M Eckhardt and R Gerardy Schahn, unpublished data).

An aliquot of 200 µl of Tris-buffered incubation medium containing 0.5% Tween 20 (pH 7.0) and 20 µl of sample or standard was filled into tubes coated with MAb 735 and incubated for 1 h at room temperature. After washing, 200 µl of MAb BW SCLC-1 conjugated to an acridinium N-acylsulfonamide label was added to each well. After incubation for 1 h at room temperature, the reaction was terminated by an additional washing cycle. The chemiluminescence activity was determined by using the BeriLux Analyser 250 (Behringwerke, Marburg, Germany). The results were expressed as kU $l^{-1}$ as previously described (Takamatsu et al, 1994). All measures were performed at least twice for internal control.

*Received 29 September 1997*
*Revised 5 December 1997*
*Accepted 11 December 1997*

*Correspondence to:* S Glüer

**Table 1** Clinical data and serum polysialylated NCAM levels of 14 children with different grades and stages of neuroblastoma

| No. | Patient | Histological grade[a] | Stage[b] | *N-myc* amplification | Polysialylated NCAM (kU $l^{-1}$) | Immunohisto-chemistry[c] | Clinical course |
|---|---|---|---|---|---|---|---|
| 1 | F. 2 months | Neuroblastoma grade 3 | 4 | nd | **1377.0** | ND | Died of disease |
| 2 | F. 7 months | Neuroblastoma grade 3 | 4 | 25 | **301.3** | +++ | Died of disease |
| 3 | M. 2 years 1 month | Neuroblastoma grade 3 | 3 | 30 | **280.6** | +++ | Died of disease |
| 4 | F. 3 years 1 month | Neuroblastoma grade 3 | 3 | 40 | **264.9** | +++ | Still under therapy |
| 5 | F. 6 months | Neuroblastoma grade 3 | 4S | 1 | **255.4** | +++ | Disease-free 12 months |
| 6 | M. 6 months | Neuroblastoma grade 3 | 1 | 1 | **221.7** | +++ | Disease-free 9 months |
| 7 | F. 1 year 1 month | Ganglioneuroblastoma grade 1a | 2b | 1 | **128.5** | + | Disease-free 9 months |
| 8 | F. 4 years 5 months | Ganglioneuroblastoma grade 1a | 3 | 1 | **90.2** | + | Disease-free 18 months |
| 9 | F. 4 months | Neuroblastoma grade 3 | 1 | 1 | 52.9 | +++ | Disease-free 3 months |
| 10 | F. 1 month | Neuroblastoma grade 3 | 1 | 1 | 51.5 | ++ | Disease-free 3 months |
| 11 | F. 4 years 3 months | Ganglioneuroblastoma grade 1b | 1 | 1 | 20.4 | ND | Disease-free 10 months |
| 12 | M. 8 years 2 months | Ganglioneuroblastoma grade 1b | 1 | 1 | 15.0 | – | Disease-free 14 months |
| 13 | M. 1 year 2 months | Ganglioneuroblastoma grade 1a | 1 | 1 | 38.8 | + | Disease-free 16 months |
| 14 | F. 5 years 4 months | Ganglioneuroblastoma grade 1b | 1 | 1 | 30.7 | – | Disease-free 6 months |

Bold numbers indicate pathological levels. F. female: M. male: ND. not done: –. no staining: +. few (<1/3 cells): ++. a lot (>1/3 cells): +++. most (>2/3 cells) stained. [a]According to Hughes et al (1974). [b]According to Brodeur et al (1988). [c]Immunoreactivity for polysialylated NCAM expressed in tumour cells stained/total tumour cells.

**Figure 1** Serum levels of polysialylated NCAM in 269 healthy control subjects. Mean value 26.8 kU $l^{-1}$. range 4.4–62.9 kU $l^{-1}$.KU $l^{-1}$ were defined as described previously (Takamatsu et al. 1994)

**Figure 2** Lymph node metastasis of patient no. 2 with a neuroblastoma grade 3. stage 4. Immunohistochemical staining with MAb 735. showing strong expression of polysialylated NCAM on the tumour cells. Arrowhead indicates lymphocytes. negative for polysialylated NCAM (original magnification ×66)

### Immunohistochemistry

Cryostat sections (5 μm) of snap-frozen tumour specimens were further processed for immunohistochemistry using the APAAP technique according to Cordell et al (1984). Briefly. the slides were fixed in ice-cold acetone for 10 min and air dried. After preincubation with normal rabbit serum. the MAb 735 (Frosch et al. 1985) was added for 1 h at room temperature. After washing. the slides were incubated with rabbit anti-mouse immunoglobulin and the APAAP complex twice for 1 h each. Colour development was obtained with Naphthol-AS-Biphosphate and New Fuchsin. Positive reaction of the counterstained cells resulted in bright red staining of the cytoplasm. Controls included the omission of primary and/or secondary antibodies. preincubation of the sections with bacteriophage endoneuraminidase for 2 h at 37°C. which has been shown previously to recognize specifically and degrade polysialic acid (Gerardy-Schahn et al. 1995). and staining with the NCAM-specific MAb UJ13A (Patel et al. 1989).

**Figure 3** Ganglioneuroblastoma of patient no. 12. Almost no reactivity for polysialylated NCAM in immunohistochemistry with MAb 735 (original magnification ×66)

### RESULTS

Mean value of the 269 healthy control subjects was 26.8 kU $l^{-1}$. As shown in Figure 1. the values ranged from 4.4 to 62.9 kU $l^{-1}$. There

**Figure 4** Follow-up polysialylated NCAM levels during therapy: serum concentrations decreased in patients with high serum levels initially and remained low in patients with normal levels. Note variable scale was no correlation with age or sex of the children. In this study, all values above 60 kU $l^{-1}$ were designated as pathological.

The data of the neuroblastoma patients are summarized in Table 1: two infants (nos 1 and 2) with stage 4 disease and one child (no. 3) with stage 3 disease had excessively high (1377.0 kU $l^{-1}$) or high serum levels (301.3, 280.6 kU $l^{-1}$ respectively). These children died of rapid tumour progression despite intensive chemotherapy. Patient no. 4 with stage 3 disease and an initial serum level of 264.9 kU $l^{-1}$ is currently still receiving chemotherapy. One infant with stage 4S disease (no. 5) had an initial serum level of 255.4 kU $l^{-1}$. Another child (no. 6) with a large stage 1 neuroblastoma of histological grade 3 (tumour weight 200 g, the tumour was limited to one adrenal gland) had a serum level of 221.7 kU $l^{-1}$. Two infants with small adrenal stage 1 neuroblastoma (nos 9 and 10, tumour weight 9.5 g and 4.5 g respectively) had serum levels within normal ranges. Both children with more differentiated tumour types but extensive disease (nos 7 and 8) had moderately raised levels (128.5, 90.2 kU $l^{-1}$ respectively). All children with ganglioneuroblastoma stage 1 (nos 11–14) had normal serum levels (20.4, 15.0, 38.8, 30.7 kU $l^{-1}$ respectively) at diagnosis.

N-*myc* gene amplification (25- to 40-fold) was found in the patients with the highest serum levels (nos 2–4), whereas no amplification of the gene was found in the other tumours (Table 1). Immunohistochemistry revealed high expression of polysialylated NCAM in the tumour specimens of patients nos 2–6 and no. 9 (Table 1 and Figure 2), whereas the specimens of the other patients had considerably smaller amounts of polysialylated NCAM (Table 1 and Figure 3).

As shown in Figure 4, eight children were available for follow-up studies. In patients with high levels initially, the serum concentrations

 © Cancer Research Campaign 1998

of polysialylated NCAM decreased during therapy. After reaching normal levels, values remained normal during follow-up. In children with low levels initially, follow-up studies did not reveal any changes in serum concentrations.

### DISCUSSION

In adult patients, serum polysialylated NCAM levels above 20 kU $l^{-1}$ have been shown to be pathological (Jaques et al, 1993; Kaiser et al, 1994). We found levels of up to 62.9 kU $l^{-1}$ in healthy children in whom the levels are on average higher (mean 26.8 kU $l^{-1}$) than in adults. As the values cover a wide range (from 4.4 to 62.9 kU $l^{-1}$) we could not establish an age-dependent decrease in serum polysialylated NCAM. This is in contrast to previous findings in cerebrospinal fluid when a decrease was observed during the first year of life (Weisgerber et al, 1990).

The mechanism by which NCAM forms appear in the serum is not known. The fact that there seems to be a relationship between the serum levels and the estimated amount of polysialylated NCAM-positive cells may indicate a constant release of this molecule from the cell surface. PSA-NCAM is expressed also on natural killer cells and a subset of T lymphocytes (van Riet et al, 1991). Theoretically, reactive shifts within these populations may be of relevance to the PSA-NCAM serum level. However, the serum levels of PSA-NCAM are low initially and during the course of most other malignant and benign childhood diseases. Moreover, we found a strong correlation between the levels of PSA-NCAM and the number of viable cells in cultures of neuroblastoma cells, indicating that the molecule in fact derives from the tumour cells (unpublished observations).

In a recent study (Figarella-Branger et al, 1996), polysialylated NCAM levels in cerebrospinal fluid were shown to correlate with clinical stage and outcome of patients with medulloblastoma. Our data show that polysialylated NCAM is a useful marker in the far more easily accessible serum of children suffering from neuroblastoma.

A reliable and widely accepted prognostic factor in childhood neuroblastoma is amplification of the N-*myc* gene in the tumour (Brodeur et al, 1984). In our study, N-*myc* gene amplification was found in the three children with the highest serum levels of polysialylated NCAM, two of whom died subsequently of progressive disease. In contrast, amplification was not present in the tumours of the patients with moderately raised or normal serum levels. However, two children with non-amplifying tumours had high (>200 kU $l^{-1}$) serum levels, one with stage 4S disease and one with a large stage 1 adrenal tumour. Obviously, serum polysialylated NCAM did not distinguish these children from the others with a poor prognosis. As a consequence, it seems possible to speculate that, rather than indicating a defined tumour stage, serum polysialylated NCAM levels reflect the total amount of polysialylated NCAM-positive tumour cells, which in turn represent the viable tumour mass.

Polysialylation of NCAM has previously been shown to modulate the adhesive functions of tumour cells and to enhance their invasive and metastatic potential (Yang et al, 1992). Therefore, it is conceivable that high serum levels of polysialylated NCAM indicate more malignant tumour types. In our study, significantly lower serum levels were found in children with differentiated tumour types compared with undifferentiated tumours. In differentiated ganglioneuroblastomas, pathological levels occurred only in children with advanced stages of disease. Thus, serum polysialylated NCAM levels are influenced by both the tumour mass and histological differentiation. Serum levels decreased during successful therapy and remained low in patients during remission. This indicates that soluble polysialylated NCAM may be a useful marker for maintaining therapy in neuroblastoma patients.

### ACKNOWLEDGEMENTS

Supported by the Deutsche Forschungsgemeinschaft, Grant GL 173/2-1 (SG) and by the Boehringer Mannheim Research Fund (RG-S).

### REFERENCES

Brodeur GM, Seeger RC, Schwab M, Varmus HE, Bishop JM (1984) Amplification of N-myc in untreated human neuroblastomas correlates with advanced stages of disease. *Science* **224**: 1121–1124

Brodeur GM, Seeger RC, Barrett A, Berthold F, Castleberry RP, D'Angio G, Bernardi B, Evans AE, Favrot M, Freeman AI, Haase G, Hartmann O, Hayes FA, Helson L, Kemshead J, Lampert F, Ninane J, Ohkawa H, Philip T, Pinkerton CR, Pritchard J, Sawada T, Siegel St, Smith EI, Tsuchida Y, Voute PA (1998) International criteria for diagnosis, staging, and response to treatment in patients with neuroblastoma. *J Clin Oncol* **6**: 1874–1881

van Camp B, Durie BGM, Spier C, de Waele M, van Riet I, Vela E, Frutiger Y, Richter L, Grogan TM (1990) Plasma cells in multiple myeloma express a natural killer cell-associated antigen: CD 56 (NHK 1; Leu 19). *Blood* **76**: 337–382

Cordell JL, Falini B, Erber WN, Ghosh AK, Abdulaziz Z, MacDonald S, Pulford KAF, Stein H, Mason DY (1984) Immunoenzymatic labeling of monoclonal antibodies using immune complexes of alkaline phosphatase and monoclonal anti-alkaline phosphatase. *J Histochem Cytochem* **32**: 219–229

Figarella-Branger D, Dubois C, Chauvin P, DeVictor B, Gentet J-C, Rougon G (1996) Correlation between polysialic-neural cell adhesion molecule levels in CSF and medulloblastoma outcomes. *J Clin Oncol* **14**: 2066–2072

Frosch M, Görgen I, Boulnois GJ, Timmis KN, Bitter-Suermann D (1985) NZB mouse system for production of monoclonal antibodies to weak bacterial antigens: isolation of an IgG antibody to the polysaccharide capsules of *Escherichia coli* K1 and group B meningococci. *Proc Natl Acad Sci USA* **82**: 1194–1198

Gerardy-Schahn R, Bethe A, Brennecke T, Mühlenhoff M, Eckhardt M, Ziesing S, Lottspeich F, Frosch M (1995) Molecular cloning and functional expression of bacteriophage PK1E-encoded endoneuraminidase Endo NE. *Mol Microbiol* **16**: 441–450

Häyrinen J, Bitter-Suermann D, Finne J (1989) Interaction of meningococcal group B monoclonal antibody and its Fab fragment with α-2-8-linked sialic acid polymers: requirement of a long oligosaccharide segment for binding. *Mol Immunol* **26**: 523–529

Hughes M, Marsden HB, Palmer MK (1974) Histologic pattern of neuroblastoma related to prognosis and clinical staging. *Cancer* **34**: 1706–1711

Jaques G, Auerbach B, Pritsch M, Wolf M, Madry N, Havemann K (1993) Evaluation of serum neural cell adhesion molecule as a new tumor marker in small cell lung cancer. *Cancer* **72**: 418–425

Kaiser U, Jaques G, Havemann K, Auerbach B (1994) Serum NCAM: a potential new prognostic marker for multiple myeloma. *Blood* **83**: 871–873

Ledermann JA, Pasini F, Olabiran Y, Pelosi G (1994) Detection of the neural cell adhesion molecule (NCAM) in serum of patients with small-cell lung cancer (SCLC) with 'limited' or 'extensive' disease, and bone-marrow infiltration. *Int J Cancer Suppl* **8**: 49–52

Moolenaar CEC, Muller EJ, Schol DJ, Figdor CG, Bock E, Bitter-Suermann D, Michalides RJAM (1990) Expression of neural cell adhesion molecule related sialoglycoprotein in small cell lung cancer and neuroblastoma cell lines H69 and CHP-212. *Cancer Res* **50**: 1102–1106

Patel K, Rossell RJ, Boune S, Moore SE, Walsh FS, Kemshead JT (1989) Monoclonal antibody UJ13A recognizes the neural cell adhesion molecule (NCAM). *In J Cancer* **44**: 1062–1068

van Riet I, de Waele M, Remels L, Lacor R, Schots R, van Camp B (1991) Expression of cytoadhesion molecules (CD56, CD54, CD28 and CD29) by myeloma plasma cells. *Br J Haematol* **79**: 421–427

© Cancer Research Campaign 1998

Rougon G (1993) Structure, metabolism and cell biology of polysialic acids. *Eur J Cell Biol* **61**: 197–207

Smith SR, Auerbach B, Morgan L (1996) Serum neural cell adhesion molecule in multiple myeloma and other plasma cell disorders. *Br J Hematol* **92**: 67–70

Takamatsu K, Auerbach B, Gerardy-Schahn R, Eckhardt M, Jaques G, Madry N (1994) Characterization of tumor-associated neural cell adhesion molecule in human serum. *Cancer Res* **54**: 2598–2603

Weisgerber Ch, Husmann M, Frosch M, Rheinheimer C, Peuckert W, Görgen I, Bitter-Suermann D (1990) Embryonic neural cell adhesion molecule in cerebrospinal fluid of younger children: age dependent decrease during the first year. *J Neurochem* **55**: 2063–2071

Yang P, Yin X, Rutishauser U (1992) Intercellular space is affected by the polysialic acid content of NCAM. *J Cell Biol* **116**: 1487–1496

© Cancer Research Campaign 1998